# Chagas Disease and Breast-feeding

**DOI:** 10.3201/eid1910.130203

**Published:** 2013-10

**Authors:** Francesca F. Norman, Rogelio López-Vélez

**Affiliations:** Ramón y Cajal Hospital–Instituto Ramón y Cajal de Investigación Sanitaria, Madrid, Spain

**Keywords:** Chagas disease, Trypanosoma cruzi, breast-feeding, neglected tropical disease, trypomastigotes, parasites, transmission, congenital transmission, parasitic diseases

## Abstract

Chagas disease (infection by the protozoan *Trypanosoma cruzi*) is a major parasitic disease of the Americas and one of the main neglected tropical diseases. Although various routes of transmission sre recognized, the risk for transmission of the infection through breast-feeding has not clearly been established. We reviewed the literature on transmission of *T. cruzi* through breast-feeding to provide breast-feeding mothers with Chagas disease with medical guidance. Although data from animal studies and human studies are scarce, we do not recommend that mothers with Chagas disease discontinue breast-feeding, unless they are experiencing the acute phase of the disease, reactivated disease resulting from immunosuppression, or bleeding nipples. In these cases, thermal treatment of milk before feeding the infant may be considered.

## Abstract 2

Mothers with this disease should continue breast-feeding unless they are experiencing the acute phase, reactivated disease, or bleeding nipples.

Chagas disease (infection by the protozoan *Trypanosoma cruzi*) is a major parasitic disease in the Americas and one of the main neglected tropical diseases. *T. cruzi* is transmitted to humans primarily through the infected feces of triatomine bugs that contain metacyclic trypomastigotes (vectorial transmission). Other modes of transmission include transmission through infected blood and organs, vertical transmission, oral transmission (through consumption of contaminated food or drink), and transmission through laboratory accidents. The estimated number of infections in North, South, and Central America fell from ≈20 million in 1981 to ≈8–10 million in 2005; the reduction is mainly attributed to vector control campaigns and screening of blood donations in disease-endemic areas (*1*). However, the increase in mobile populations has led to the emergence of Chagas disease in non–disease-endemic areas where vectors are noncompetent and where the infection may be transmitted by other routes. A considerable proportion of migrants from Latin America are women, which turns the focus to preventing possible transmission from infected mother to child. The risk for congenital transmission from an infected mother may range from 0.13% to 17%, and the likelihood of congenital infection appears to correlate with maternal parasite density (*2*,*3*). Screening is recommended during pregnancy to detect mothers who may transmit *T. cruzi* to the fetus, even though no specific means of preventing congenital infection during pregnancy are available (*4*). The possibility of transmission through breast-feeding may be particularly relevant because, if breast-feeding is a route for transmission after delivery, such transmission may be preventable. Although some early publications identify breast-feeding as a possible route for *T. cruzi* infection, such data are scarce, and few animal studies exist (*5*–*8*).

Apart from other benefits, exclusive breast-feeding is the ideal way to provide nutrition during the first 6 months of life (*9*), and interruption of breast-feeding in resource-poor settings does not seem feasible unless the risks clearly outweigh possible benefits. If transmission occurs, treatment of acute infections is more successful in infants and children, and the treatment is also better tolerated (i.e., causes fewer side effects) in infants and children (*10**,**11*). The risk for transmission of infection through breast-feeding and whether any means are available to minimize this risk should be determined. The objective of this study was, therefore, to review the literature on transmission of *T. cruzi* through breast-feeding to adequately inform breast-feeding mothers with Chagas disease.

## Search Strategy and Selection Criteria

We searched the literature in PubMed (www.ncbi.nlm.nih.gov/pubmed), EMBASE (www.elsevier.com/online-tools/embase), and Google Scholar (http://scholar.google.com/) for all published articles until January 2013, using the terms “breast-feeding” and “Chagas,” “breastfeeding” and “*Trypanosoma cruzi*,” “milk” and “Chagas,” and “milk” and “*Trypanosoma cruzi*.” No language, age, or sex restrictions were used. Both animal and human studies were considered. Direct access to some of the older publications was not possible, and information about results and conclusions was obtained from other articles.

## Animal Studies

As early as 1913, Nattan-Larrier reported finding *T. cruzi* in the milk of laboratory animals (*12*). Disko and Krampitz found *T. cruzi* trypomastigotes in the milk of experimentally infected mice, which was not attributable to contamination of milk with blood. However, they did not subsequently demonstrate infection of suckling mice (*13*). In 1972, Miles examined the milk of mice in the acute phase of the disease and detected *T. cruzi* trypomastigotes and antibodies against *T. cruzi,* but concluded that, experimentally, even in the acute phase of the disease, transmission through breast-feeding was rare (*14*). In another study, Campos et al. found that nursing mice born to lactating females in the acute phase of the infection were not infected (*15*). Another study on vertical transmission of *T. cruzi* among chronically infected laboratory rats did not demonstrate transmission through breast-feeding, and histopathologic examination of the mammary glands of the infected rats did not reveal the parasites (*16*). More recently, Martins et al. inoculated 15 female mice with Y strain *T. cruzi* trypomastigote forms a few hours after they gave birth, and breast-feeding of offspring was continued. Despite confirmed infection in female mice, no infection through breast-feeding was documented in the 142 offspring examined (examination of fresh blood by microscopy) (*17*). Other authors have not been able to confirm experimental transmission through breast-feeding during the acute phase of the disease (*18*–*21*), and researchers have hypothesized that maternal antibodies transferred with the milk may play a protective role (*14*,*22*).

Studies that examined pasteurization and microwave treatment of human milk to prevent transmission of Chagas disease have demonstrated transmission when human milk contaminated with Y strain trypomastigotes was orally administered to mice. However, such transmission occurred under experimental conditions, not natural breast-feeding, and the milk was artificially contaminated. In 1 experiment, batches of laboratory mice were orally or intraperitoneally inoculated with human milk that was contaminated with *T. cruzi* trypomastigotes and pasteurized, contaminated with *T. cruzi* and not pasteurized, or not contaminated with *T. cruzi*. Only mice that were given contaminated nonpasteurized milk (orally or intraperitoneally) were infected (detected by examination of peripheral blood) (*23*). In a similar study, mice that received either infected or noninfected microwave-treated milk (63°C, domestic microwave oven 2,450 MHz, 700 W), orally or intraperitoneally, were not infected (negative parasitologic and serologic results), whereas mice that received contaminated milk that had not been microwaved acquired the infection (*24*).

Studies involving metacyclic trypomastigotes from triatomine feces have found that contaminated milk seems to be an adequate medium (when compared with other types of food and water) for oral transmission of *T. cruzi* to mice, and this fact has been attributed to its moisture and nutrient content (*25*). In certain animal experiments, infection through the oral route (not breast-feeding) has been established by using blood trypomastigotes, and the success of this mode of transmission appeared dependent on the size of the inoculum used (*26*,*27*). Transmission of *T cruzi* by breast-feeding is possible in animal models, but oral infections may be difficult to establish unless metacyclic forms of the parasite are involved.

## Studies in Humans

Two reports from 1936 and 1983 describe finding trypomastigotes in the milk of mothers in the acute phase of Chagas disease. Mazza et al. (*5*) reported transmission through lactation; in the other study, the newborn was not breast-fed (*28*). However, another publication later indicated that in the study by Mazza et al., the collected milk was contaminated by blood (*29*). Medina-Lopes reported 2 cases of acute Chagas disease in infants that had been acquired through breast-feeding from mothers in the chronic phase. In 1 of these cases, no parasites were found in the milk, and the source of the infection was postulated to be infected blood because the mother had bleeding nipples. In the other case, congenital, vector-borne, and transfusion-associated transmission were excluded, and transmission was suspected to have occurred through breast-feeding (*6*,*7*).

In 1988, Bittencourt et al. were unable to demonstrate transmission of infection through breast-feeding. Samples of milk/colostrum from 78 mothers in the chronic phase of the disease were studied (5 mothers had parasitemia at the time of milk collection). Results of the following were negative: the parasitologic study of all sample-inoculated mice and the serologic tests of serum samples from 97 breast-fed children born free of infection (*22*).

In 1990, Shikanai-Yasuda et al. published a series of reports of cases of acute Chagas disease, and breast-feeding was considered a possible route of transmission for 2 patients (*30*). Amato Neto et al. (*31*) could not document the presence of *T. cruzi* in the colostrum and breast milk of 40 women with Chagas disease through direct observation, culture, and inoculation. The possibility of using a larger study population and/or more efficient diagnostic techniques was highlighted (*31*).

Other studies that examined maternal transmission of *T. cruzi* included breast-feeding as a possible contributing factor, but once again, this mode of transmission could not be demonstrated. In 2004, Rassi et al. found 2 cases of maternal transmission of infection in 2 patients who had also been breast-fed, but this mechanism could not be established, and the infections may have resulted from vertical transmission (*32*).

The data on transmission of *T. cruzi* through breast-feeding in humans are scarce; reports are not recent and the findings have several limitations. Additional data on methods used to diagnose infection and exclude other forms of transmission in these studies are summarized in the Table.

**Table:** Summary of human studies on transmission of *Trypanosoma cruzi* through breast-feeding*

Reference	Phase of infection, mother	Methods and findings	Comments
Mazza et al, 1936 (*5*)	Acute	No trypomastigotes detected by direct methods in newborn at d 10. Parasites detected at 3 mo when acute Chagas disease diagnosed in newborn and concomitantly found by direct methods in mothers´ milk	Congenital transmission not adequately ruled out. Detection of parasites in milk may have been due to contamination with maternal blood containing trypomastigotes from a bleeding nipple (*29*).
Medina-Lopes and Macedo, 1983 (*28*)	Acute	Wk 5 of maternal acute infection (before delivery): direct examination of colostrum: negative; intraperitoneal inoculation of mice with colostrum and xenodiagnosis: positive. Maternal milk (after delivery) intraperitoneally inoculated into mice and xenodiagnosis: positive	Demonstrated *T.cruzi* in colostrum and milk. Contamination from blood not mentioned but unlikely as newborn did not breast-feed (assuming intact nipples during sample extraction). Congenital infection ruled out by using 4 different (unspecified) diagnostic methods
Medina-Lopes, 1983 (*6*)	Chronic	Congenital transmission excluded by unspecified methods. Vectorial transmission excluded. Acute infection diagnosed in 2-mo-old newborn by unspecified methods.	Mother had nipple bleeding: infected blood in milk may have been the source of the infection. Milk direct exam was negative.
Medina-Lopes, 1988 (*7*)	Chronic	Infection excluded at birth by modified Strout and xenodiagnoses by using cord blood. At 6 mo IFAT and IHA: positive. At 7 mo modified Strout: positive. Transfusional and vectorial transmission excluded (housing inspected for vector)	Mother had positive xenodiagnosis at time of delivery. Mother had nipple fissures 1 wk postpartum; unclear whether breast-feeding occurred at time. No examination of maternal milk performed. Older sibling not infected even though also breast-fed at the same time.
Bittencourt et al., 1988 (*22*)	Chronic	Milk/colostrum from 78 mothers (101 samples) studied by direct examination and inoculation of mice. Mice tested by direct blood examination, xenodiagnosis, and IFAT for *T. cruzi* antibodies. No mice infected. Transmission in 97 breast-fed children excluded at birth by direct blood examination-microhematocrit and xenodiagnosis and by serology (IFAT) at 6–24 mo.	5 mothers had positive blood xenodiagnosis at time of milk collection. Mothers recommended to avoid breast-feeding if nipple bleeding. Average time of breast-feeding was 7 mo; unclear whether any infant tested by serology before ending breast-feeding-
Shikanai-Yasuda et al., 1990 (*30*)	Chronic	Acute Chagas disease diagnosed at 3.5 and 9.5 mo in 2 infants, respectively, by direct examination of peripheral blood.	Vectorial transmission unlikely (urban area). Congenital transmission not investigated. Mother of the 9.5-mo-old infant had nipple fissures and bleeding during breast-feeding.
Amato Neto et al., 1992 (*31*)	Chronic	Search for *T. cruzi* in colostrum and breast milk of 40 chronically infected women through direct observation, culture, and inoculation	No evidence of *T. cruzi* in samples
Rassi et al., 2004 (*32*)	Chronic	Identified 2 cases of infection in children 2- and 5-y-old, respectively, detected by at least 2 positive serologic tests (complement fixation, IHA, ELISA, and IFAT)	Transfusional and vectorial transmission excluded. Probable congenital infections but both patients also breast-fed and contribution of this factor could not be ruled out

*IFAT, indirect immunofluorescence antibody test; IHA, indirect hemagglutination antibody test.

## Discussion

In mice, oral transmission of *T. cruzi* infection through human milk contaminated with trypomastigotes is possible, although natural transmission through breast-feeding has not been clearly demonstrated in recent studies. Infection in laboratory mice acquired after oral or intraperitoneal administration of human milk contaminated with *T. cruzi* trypomastigotes may be prevented by thermal treatment (pasteurization or microwave treatment at 63°C) of milk, which can inactivate the trypomastigotes. Such treatment of milk may therefore be an option for lactating mothers who have the acute form of the infection or bleeding nipples.

In humans, the oral route is an efficient mode of transmission for *T. cruzi*, as has been demonstrated in recent outbreaks of infection acquired through contaminated food and fruit juices (*33*–*35*). Ingestion of food or drink contaminated with vector feces (containing metacyclic trypomastigotes) or crushed insects is the most likely mode of oral infection (*36*). The concentration of the ingested inoculum may result in the different clinical manifestations and variations in attack rates reported in outbreaks (*34*).

Milk appears to be an adequate medium for transmission of *T. cruzi* because of its moisture and nutrient content and, in the case of human milk, body temperature would also be favorable (the parasite can be destroyed in dry vector feces and by high temperatures) (*23*,*24*,*36*). Despite this, except for some dated cases, in humans, transmission through breast-feeding has not been reported.

A further consideration to account for the relative lack of infections transmitted through breast-feeding is that, if they occur at all, blood-form trypomastigotes would be expected to occur in human milk and not metacyclic trypomastigotes, which are found in the vector. Blood, metacyclic, and culture trypomastigotes are similar in form and structure, but the different forms may have different surface glycoproteins, which are used for cell invasion (*17*,*37*). Metacyclic trypomastigotes may have specialized surface molecules (such as gp82, a member of the gp85/ trans-sialidase superfamily, or pepsin-susceptible gp90 isoforms, which make parasites highly invasive against target cells after contact with gastric juice) with functions for mucosal invasion that are not present in blood-form trypomastigotes. The latter, in turn, express other molecules that facilitate dissemination within the host (*38*–*40*). Even though trypomastigote forms of the Y strain of *T. cruzi*, which expresses glycoprotein 82 in metacyclic forms and should have high infectivity, were used in some experiments, transmission through breast-feeding could not be demonstrated. Blood-form trypomastigotes may therefore not be as efficient as metacyclic trypomastigotes in establishing mucosal invasion of the gastrointestinal tract (partly because of their decreased capacity to migrate through the gastric mucin layer), even though oral infections with blood trypomastigotes have been demonstrated experimentally with the possibility of infection depending on amount of inoculum. Peptic digestion may also destroy some of the infective forms, mainly blood trypomastigotes (*40*).

On the basis of the data examined, oral infection through breast-feeding does not appear to be an efficient mode of transmission for *T. cruzi*. In humans, milk contamination by *T. cruzi* has been described only in isolated cases. In the acute phase, patent parasitemia occurs, but *T. cruzi* has been demonstrated in breast milk only in 1 case (transmission did not occur because the newborn was not breast-fed) (*27*). In the chronic phase, parasitemia is usually low grade and tends to fluctuate. A couple of cases of acute infection in breast-feeding infants of mothers in the chronic phase have been reported, but transmission may have resulted from the ingestion of blood from bleeding nipples (*6*,*7*). In older studies, it is not always clear how accurately vertical and other modes of transmission had been excluded with the diagnostic tools available at the time, and in other cases, breast-feeding is contemplated only as a possible route of infection. Many of the studies reported were performed some years ago, and current novel techniques, such as PCR, may aid in detecting low parasite loads. On the other hand, new techniques may even yield equivocal results (e.g., because of the presence of infected blood but not true contamination of milk by *T. cruzi*) if contamination with infected blood were below the level of detection found with other methods. In humans, transmission of *T. cruzi* through maternal milk (not contaminated with infected blood) may not be considered clearly proven.

As previously postulated in laboratory studies, in humans, a combination of several factors likely account for the low efficiency of breast-feeding as a possible mode of transmission of *T. cruzi* infection. These include the low number of trypomastigotes ingested by the lactating infant, the biologic forms and strains of *T. cruzi* involved, the passage of protective maternal antibodies, and the destruction of some parasites by gastric juice.

A limitation of this review was that some of the articles dated back several decades and were not readily available. Some information about these studies was obtained from other articles.

Published studies on the transmission of *T. cruzi* through breast-feeding are scarce. After reviewing the various studies, and given the demonstrated benefits of breast-feeding (especially in lower income disease-endemic countries where a safe and inexpensive alternative may not be available), we conclude that discontinuing or interrupting breast-feeding by mothers with chronic Chagas disease is not recommended. If the mother has fissures or bleeding nipples, temporary discontinuation of breast-feeding may be recommended, although thermal treatment (pasteurization or microwaving) of expressed milk before feeding the infant may be a safe alternative. When mothers are in the acute phase of the disease (an infrequent situation that will occur almost exclusively in disease-endemic countries) or have reactivated disease (resulting from immunosupression, in which circulating parasitemia level may be high), breast-feeding may pose a risk for the infant. The benefits of using thermally treated expressed milk should be considered on an individual basis. To avoid any possibility of transmission, human milk banks should exclude mothers with Chagas disease as donors. As other modes of transmission of Chagas disease come under control (vector-borne transmission and transmission through infected blood/organs), further studies are needed on the possibility of transmission through breast-feeding, as this could occur even in countries where the disease is nonendemic. 
